# Cutaneous Vasodilation during Local Heating: Role of Local Cutaneous Thermosensation

**DOI:** 10.3389/fphys.2016.00622

**Published:** 2016-12-20

**Authors:** Gary W. Mack, Kristopher M. Foote, W. Bradley Nelson

**Affiliations:** ^1^Department of Exercise Sciences, The Human Performance Research Center, Brigham Young UniversityProvo, UT, USA; ^2^Department of Anesthesiology, 1500 E Medical Center Drive, University of MichiganAnn Arbor, MI, USA; ^3^Department of Natural Sciences, Ohio Dominican UniversityColumbus, OH, USA

**Keywords:** neurogenic vasodilation, axon reflex, thermosensation, transient receptor potential ion channels, skin blood flow

## Abstract

We tested the hypothesis that cutaneous vasodilation during local skin heating in humans could be manipulated based upon the ability to desensitize TRPV4 ion channels by applying the thermal stimuli in a series of pulses. Each subject was instrumented with intradermal microdialysis probes in the dorsal forearm skin and perfused with 0.9% saline at 1.5 μl/min with local skin temperature controlled with a Peltier unit (9 cm^2^) at 34°C. Local skin temperature was manipulated for 50 min in two classic ways: a step increase to 38°C (0.1°C/s, *n* = 10), and a step increase to 42°C (*n* = 10). To desensitize TRPV4 ion channels local skin temperature was manipulated in the following way: pulsed increase to 38°C (1 pulse per min, 30 s duration, 1.0°C/s, *n* = 10), and 4) pulsed increase to 42°C (1.0°C/s, *n* = 9). Skin blood flow (SkBF, laser Doppler) was recorded directly over the middle microdialysis probe and the dialysate from all three probes were collected during baseline (34°C) and each skin heating period. The overall cutaneous vascular conductance (CVC) response to local heating was estimated from the area under the % CVCmax-time curve. The appearance of the neuropeptide calcitonin gene related peptide (CGRP) in dialysate did not change with skin heating in any protocol. For the skin temperature challenge of 34 to 38°C, the area under the % CVCmax-time curve averaged 1196 ± 295 (SD) % CVCmax•min, which was larger than the 656 ± 282% CVCmax•min during pulsed heating (*p* < 0.05). For the skin temperature challenge of 34 to 42°C, the area under the % CVCmax-time curve averaged 2678 ± 458% CVCmax•min, which was larger than the 1954 ± 533% CVCmax•min during pulsed heating (*p* < 0.05). The area under the % CVCmax•min curve, was directly proportional to the accumulated local skin thermal stress (in °C•min) (*r*^2^ = 0.62, *p* < 0.05, *n* = 39). This association indicates a critical role of local integration of thermosensitive receptors in mediating the cutaneous vasodilator response to local skin heating. Given that we saw no differences in the levels of CGRP in dialysate, the role of the vasoactive peptide CGRP in the cutaneous vasodilator response to local skin heating is not supported by our data.

## Introduction

Cutaneous thermosensing, especially noxious heat or cold, is critical for survival of most organisms. Thermosensation in human skin is mediated by primary sensory afferents that detect, transduce and transmit thermal information to the central nervous system. The thermal information provided by these primary afferents drive thermoregulatory reflexes that act through the cutaneous sympathetic nervous system to alter skin blood flow and sweating, both of which impact heat transfer. In terms of thermoregulatory control there is a functional relationship between skin temperature (Tsk) and the level of skin blood flow (Wenger et al., 1986). This relationship is maintained following blockade of the cutaneous sympathetic nerves (Wenger et al., 1986). As such, thermosensation regulates skin blood flow by a combination of local and reflex mechanisms. Temperature sensing at the molecular level has been attributed to several members of the transient receptor potential (TRP) superfamily of ion channels (Clapham, 2003). Yet, we know very little about the relationship between the sensing properties of “thermo” TRP's and physiological responses (e.g., skin blood flow).

Innocuous heating of the skin is sensed by warm receptors in the skin that show activity at temperatures between 30 and 47° (Hensel and Iggo, 1971). Some warm afferents have peak discharge rates at a Tsk of ≈41°C while others continue to increase firing rate up till ≈47°C. Sensory transduction of the cutaneous warm receptors is thought to involve a subfamily of TRP ion channels that are also activated by vanilloids and are referred to as TRPV ion channels (TRPV1, TRPV2, and TRPV3) (Szallasi and Blumberg, 1999; Ramsey et al., 2006). The thermosensitivity of theses ion channels has been determined from *in vitro* whole-cell patch clamp studies and the thermosensitivity of the TRPV3 and TRPV4 ion channels appear quite similar to the discharge patterns of warm sensitive afferents (Konietzny and Hensel, 1977). In knock-out mice (TRPV3^−/−^ and TRPV4^−/−^ mice), behavior assays of thermosensitivity have shown altered temperature preference compared to their wild-type cohort (Todaka et al., 2004; Caterina, 2007). While this data supports the role of thermo TRP's in cutaneous thermosensation, the altered temperature preference in these knock-out mice appears highly dependent on the genetic background and gender of the mouse model (Huang et al., 2011). Another problem with interpreting these data is that a change in an animal's temperature preference cannot be attributed solely to altered thermosensation in the primary sensory neurons because the processing of thermal information in the central nervous system is also affected. One differentiating characteristic of the TRPV3 and TRPV4 ion channels is their response to repetitive thermal stimulus. TRPV3 ion channels show sensitization (Xu et al., 2002) while TRPV4 ion channels show desensitization to repetitive thermal stimulus (Güler et al., 2002; Chung et al., 2004). In the present study we used non-pharmacological approach to identify the role of TRP channels because of a lack of safe and selective TRP channel blocking agents for human use. We used a pulsed heating protocol to try to desensitize TRPV4 ion channels and produce an attenuated skin blood flow response.

A noxious thermal stimuli delivered to the skin (Tsk > 43°C), detected as heat pain, is thought to be monitored by TRPV1 and transient receptor potential melastatin-3 (TRPM3) containing sensory neurons. Both TRPV1 and TRPM3 ion channels are expressed in cutaneous sensory nerves at similar levels. TRPM3^−/−^ mice show a strong deficit in detection of noxious heat (Vriens et al., 2011) but unaltered temperature preference. However, TRPV1^−/−^ mice demonstrate altered behavioral thermoregulation (Caterina, 2007) indicating a potential role of the TRPV1 ion channel in both thermosensation and heat pain. Of specific interest is that a portion of the skin blood flow response to innocuous heating is blunted by blocking the TRPV1 ion channel with capsazepine (Wong and Fieger, 2010). The increase in skin blood flow via TRPV1 ion channels is consistent with the activation of an axon-reflex and the release of vasoactive neuropeptides, presumably calcitonin gene-related peptide (CGRP) and Substance P (Brain and Williams, 1988; Brain et al., 1993). Both TRPV1 and TRPM3, when activated by a ligand (capsaicin and neurosteriod pregnenolone, respectively) produce an axon reflex mediated release of CGRP (Brain et al., 1986; Holzer, 1991; Tominaga and Caterina, 2004; Held et al., 2015). Unfortunately, there is no data indicating that activating TRPV1 ion channels in human skin during local thermal stimuli actually results in an increase in local CGRP concentration.

Based upon published (Gifford et al., 2012) and additional preliminary work in our laboratory, we designed a series of experiments in an attempt to address the potential contribution of TRPV1, TRPV3, and TRPV4 ion channels to the increase in skin blood flow during a local thermal stimulus in human skin. First, we compared the vasodilatory effect of a step change in Tsk from 34 to 38°C to that achieved when the same temperature differential was delivered in repetitive 30 s pulses. If TRPV4 ion channels played an important role in the cutaneous blood flow response, then the repetitive thermal stimuli should desensitize TRPV4 ion channels and blunt the vasodilator response. On the other hand, if TRPV3 ion channels were important we would expect a sensitization of the ion channel and an augmented cutaneous blood flow response. Second, we also compared the vasodilatory effect of a step change in Tsk from 34 to 42°C to that achieved when the same temperature differential was delivered in repetitive 30 s pulses. This range of local Tsk would be expected to activate TRPV1 ion channels and stimulate the release of neuropeptides. An increase in CGRP in the intradermal interstitial fluid would provide direct support for the activation of TRPV1 ion channels. We also considered that the pulsed heating protocol might provide a more robust activation of the TRPV1 ion channels.

## Methods

**Figure 1:**
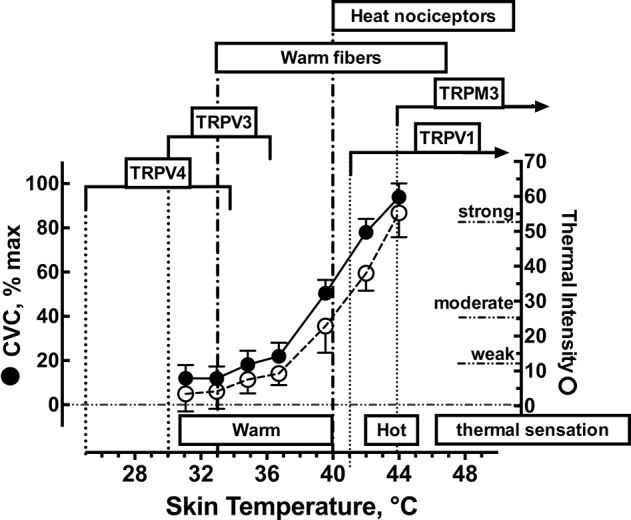
**Functional relationship between local skin temperature, cutaneous vascular conductance (CVC), and ratings of thermal sensation in human skin**. Approximate temperature spans of the transient receptor potential vanilloid (TRPV) heat receptors based on the reported thermal sensitivities confirmed in whole-cell patch clamp recordings. Temperature spans of cutaneous thermoreceptors are based upon microneurographic measurements of afferent nerve traffic and local skin temperature. Values for CVC and ratings of thermal sensations are represented as mean ± 1 SD for *n* = 12 subjects.

Initially we performed a simple preliminary study to further characterize the ratings of local cutaneous thermal sensation and skin blood flow over a wide range of local Tsk. Twelve college age subjects provided written informed consent before participating in this preliminary study. All trials were conducted in the seated position in a thermoneutral environment (27.0 ± 0.3°C). The following local skin temperatures were used: 31, 33, 35, 37, 40, 42, and 44°C. Each Tsk was applied for 20 min. Subjects rated the thermal sensation on a visual analog scale (0 to 140 mm)(Green et al., 1993) and selected as many of the following terms needed to describe the thermal sensation associated with a given skin temperature: Nothing, Cool, Cold, Warm, Hot, Burning/Stinging, Aching, or Painful. We combined this data with earlier published data (Gifford et al., 2012) to produce Figure 1 which illustrates the average skin blood flow (expressed as % of the maximal cutaneous vascular conductance, CVC) and the rating of thermal sensation from the visual analog scale during the final minute of skin heating. The purpose of this preliminary study was to define the relationship between ratings of perceived cutaneous thermosensitivity, skin blood flow and local Tsk. In addition, in Figure 1 we overlay the reported temperature ranges of thermosensitive afferents in the skin (warm fibers and heat nociceptors) and the thermosensitivities of the TRPV1, TRPV3, TRPV4, and TRPM3 ion channels. Based upon this preliminary data we chose 34°C as our baseline temperature because all subjects identified this Tsk as a weak but warm sensation. We then chose 38°C as our target Tsk to stimulate primarily cutaneous warm fibers and 42°C to activate both warm fibers and heat nociceptors.

In the follow-up study, 39 healthy subjects (20 males, 19 females; 24 ± 2 years, 170.8 ± 2.9 cm, 69.4 ± 3.6 kg) volunteered. All subjects provided written informed consent before testing and all procedures were approved by the Institutional Review Board at Brigham Young University. Before any experiment each subject was familiarized with the experimental procedures. Subjects were randomly assigned to one of four different local skin heating protocols. Protocol (#1) Single step change in local skin temperature from 34 to 38°C, rate of change = 0.1°C • s^−1^ (*n* = 10); Protocol (#2) Single step change in local skin temperature from 34 to 42°C, rate of change = 0.1°C • s^−1^ (*n* = 10); Protocol (#3) Pulsed heating, 1 pulse per min, each pulse represented a step change in local skin temperature from 34 to 38°C, rate of change = 1.0°C • s^−1^, 50% duty cycle (*n* = 10): Protocol (#4) Pulsed heating, 1 pulse per min, each pulse represented a step change in local skin temperature from 34 to 42°C, rate of change = 1.0°C • s^−1^,50% duty cycle (*n* = 9). The choice of using one pulse every minute is based upon the work of Chung and Caterina (2007) and Güler et al. (2002). All experiments were conducted with the subject seated in the upright position in a temperature controlled room at 27.5 ± 0.4 °C at the same time of day (≈ 9:00 am). Female subjects were tested during the first 10 days of the start of their menstrual cycle. Each experimental trial consisted of insertion of intradermal microdialysis probes, a 120 min recovery period, a 50 min baseline period at a Tsk = 34°C, a 50 min of skin heating based upon their respective protocol and a 40 min period in which the intradermal microdialysis probe was perfused with 28 mM sodium nitroprusside to produce maximal skin blood flow. We instrumented each subject with three linear intradermal microdialysis probes, a single 3 cm × 3 cm Peltier skin heating element, and laser Doppler flow probe on the dorsal aspect of the forearm.

Under sterile conditions, three microdialysis probes were placed intradermally in a parallel manner using a 27-gauge needle without local anesthetic. The entrance and exit sites on the skin were separated by 3.0 cm and the hollow fiber (regenerated cellulose, 18 kD molecular weight cut-off) dialysis section of each probe was 2.5 cm in length. The distance between parallel intradermal microdialysis probes was ≈ 5 mm. Each microdialysis probe was fed through the inside of the needle. The needle was then removed with the probe left within the dermis. After the placement of the probes, each subject began a 120 min recovery period in order to allow the local skin blood flow to return to baseline levels. During this period the probes were flushed with 0.9% saline at a rate of 5 μl/min with a micro-infusion pump (PHD 2000, Harvard Apparatus, MA).

**Figure 2:**
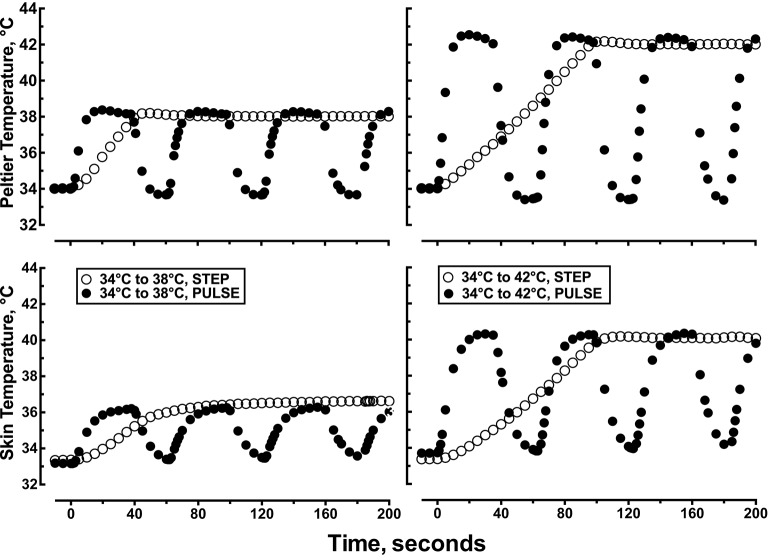
**Representative data showing the first 200 s of the time course of changes in Peltier and skin temperature during: (1) a single step change in local skin temperature from 34 to 38°C, (2) a single step change in local skin temperature from 34 to 42°C, (3) pulsed heating, 1 pulse per min, each pulse represented a step change in local skin temperature from 34 to 38°C, rate of change = 1.0°C •s^−1^, 50% duty cycle, or (4) pulsed heating, 1 pulse per min, each pulse represented a step change in local skin temperature from 34 to 42°C, rate of change = 1.0°C •s^−1^, 50% duty cycle**.

After 60 min of recovery, a computer regulated Peltier unit with a central probe holder for a flow probe was placed directly over the three microdialysis probes and regulated at the initial local starting temperature of 34.0°C. The heat transfer characteristics of the Peltier module to the skin and intradermal space was determined in pilot experiments. In those pilot experiments, skin temperature was measured with a thermocouple placed between the Peltier module and the skin and intradermal temperature was measured with a thermocouple place ≈1–2 mm into the dermis using a 25 g needle directly under the Peltier module. The skin and intradermal temperature tracked the Peltier set temperature in a linear manner (*r*^2^ = 0.94) with a slope = 0.990 and y-intercept = 0. As such, we chose to monitor and report local skin temperature under the Peltier module during all protocols. Figure 2 illustrates how the measured Tsk tracked the changes in Peltier temperature during each heating protocol.

Skin blood flow was measured by laser Doppler velocimetry using a DP7a/T probe consisting of 8 collecting fibers on a 2 mm ring with a central delivery fiber (FloLAB, Moor Instruments, UK). Data were continuously digitized (20 Hz) and stored in data files using a computerized data-acquisition system (PowerLab 16e, AD Instruments, Australia). Blood pressure was measured on the opposite arm using a non-invasive brachial artery blood pressure monitor (Colin STBP Model 780, Japan) and recorded once every 5 min during the entire experimental protocol.

120 min after microdialysis probe insertion, perfusion rate of 0.9% saline was reduced to 1.5 μl/min. The dialysate from all three probes were pooled into a single Eppendorf tube and collected every 25 min during the 50 min baseline period at Tsk = 34°C. After collection of the two baseline samples the skin heating protocol was initiated and again dialysate samples were collected once every 25 min during the 50 min skin heating protocol. Each dialysate sample was immediately frozen on dry ice and stored at −80°C for later analysis of CGRP. Interstitial dialysate levels of CGRP (CGRP_dialysate_) were measured using a high sensitivity enzyme linked immunoassay (SpiBio, Enzyme Immunoassay Kit A05481). In our hands the inter assay coefficient of variation for a quality control sample with an average CGRP concentration of 173 pg/ml was 11% and the limit of detection was 2 pg/ml.

The *in vitro* recovery properties of our microdialysis probes (*n* = 8) were determined by calculating extraction fraction (Chaurasia et al., 2007). The hollow fiber portion of each probe was placed in a shallow vessel and bathed in 2 ml of 0.9% saline. The bath was well mixed with a micro-stir bar and the microdialysis probe was perfused at 1.5 μl/min with 0.9% saline. After a 60 min equilibration period a known quantity of I^125^-labeled CGRP was added to the bath. Dialysate and bath samples (50 μl) were collected every 20 min for 100 min to establish a steady state recovery rate for each probe. The extraction fraction was calculated by dividing the activity of I^125^-labeled CGRP (dpm) in the dialysate by the activity of I^125^-labeled CGRP in the bath. The *in vitro* probe recovery averaged 7 ± 2%. We report CGRP_dialysate_ levels as the actual measured values without correcting for the probe recovery.

Cutaneous vasomotor responses to skin heating were expressed as cutaneous vascular conductance (CVC). Laser Doppler flux readings (volts) were divided by mean arterial blood pressure to calculate CVC. CVC was normalized by dividing each value by the maximal CVC and expressed as % CVCmax. CVCmax was obtained at the end of each heating protocol by perfusing the intradermal microdialysis probes with 28 mM sodium nitroprusside for 40 min.

During each protocol we identified four cutaneous vasomotor events as described in previous work (Minson et al., 2001): baseline CVC, the initial rapid peak in CVC, a nadir in CVC following the initial peak, and a plateau level of CVC at the end of the local heating protocol. We also estimated the overall cutaneous vasodilator response to heating by calculating the area under the CVC- time response curve using 15 s averages of CVC. In a similar manner we estimated overall thermal stress associated with each experimental protocol by calculating the area under the Tsk –time response curve using 1 s averages. The average Tsk for each protocol was then calculated by dividing the area under the skin temperature-time curve (°C•min) by time (50 min).

All variables, except CGRP_dialysate_, demonstrated a normal distribution based upon a Goodness-of-Fit test (Shapiro-Wik W test). CGRP_dialysate_ concentrations were compared using a non-parametric ANOVA test (Freidman test). To compare other variables during each protocol we performed a two–way mixed model ANOVA. When the ANOVA indicated a significant F value for the treatment–time interaction *post-hoc* comparisons of CVC at specific time points (0, 1, 2, 3, 4, 5, 10, 15, 20, 30, 40, and 50 min) were performed using a Tukey's minimum significant difference test (JMP12, SAS). We used one-way ANOVA for repeated measures analysis of variance to identify trends in variables during each heating protocol. Linear least squares regression was used to describe the relationship between variables. Significant was set at the *p* ≤ 0.05 level. To provide an objective measure of the magnitude of any observed effect we calculated the effect size (Cohn's *d*) and its 95% confidence interval (CI). Effect sizes for differences in levels of CGRP_dialysate_ at various time points were determined using a *post-hoc* Mann-Whitney test with the CI's determined using calculations described by Cumming and Finch (Cumming and Finch, 2001) for non-central distributions.

## Results

During the step change in the Peltier heating element from 34 to 38°C mean local skin temperature averaged 37.5 ± 1.0°C (Table 1). Mean local skin temperature during the 34 to 38°C pulsed heating protocol (50% duty cycle) averaged 35.6 ± 0.5°C which was lower than during the step change in skin temperature (*p* < 0.05). During the step change in the Peltier heating element from 34 to 42°C mean local skin temperature averaged 40.9 ± 1.2°C. Local skin temperature during the pulsed heating protocol from 34 to 42°C (50% duty cycle) averaged 37.9 ± 0.8°C which was lower than during the step change in skin temperature (*p* < 0.05). The average local skin temperature during a step change from 34 to 38°C was similar to that observed during the pulsed heating from 34 to 42°C.

**Table 1:** **Biphasic skin blood flow response parameters**.

**Parameter**	**34°C to 38°C**			**34°C to 42°C**		
	**STEP**	**PULSED**	**Cohen's d (CI)**	**STEP**	**PULSED**	**Cohen's d (CI)**
Number of subjects	10	10		10	9	
Baseline, %CVC max	20.0±6.6	23.8±8.2	−0.5 (−1.38, 0.40)	20.0±6.8	20.2±6.6	−0.03 (−0.9, 0.87)
Initial Peak, %CVC max	48.3±9.6	40.2±16.3	0.6 (−0.31, 1.47)	80.8±5.4	67.2±12.6	1.4 (0.37, 2.37)
Nadir, %CVC max	28.9±8.2	26.4±9.3	0.3 (−0.61, 1.15)	57.6±9.4^†^	37.3±6.4^†^^*^	2.5 (1.21, 3.56)
Plateau, %CVC max	52.9±10.5	39.2±12.6^*^	1.2 (0.19, 2.08)	80.7±6.9^†^	66.3±12.2^†^^*^^#^	1.5 (0.4, 2.42)
CVC AUC, % CVCmax•min	1196±295	656±282^*^	−1.9 (−2.8, −0.8)	2678±458^†^	1954±533^*^^†^^#^	−1.45 (−2.4, −0.4)
Tsk AUC, °C•min	181±9	108±7^*^	8.9 (5.6, 11.0)	324±38^†^	215±14^*^^†^^#^	2.9 (2.1, 5.1)
Mean Tsk, °C	37.5±1.0	35.6±0.5^*^	2.4 (1.17, 3.43)	40.9±1.2^†^	37.9±0.8^*^^†^^#^	2.9 (1.5, 4.0)

*CI, confidence interval; CVC, cutaneous vascular conductance expressed as %max. Tsk, skin temperature. Values are mean ± 1 SD*.

* p < 0.05 different from STEP;

† *p < 0.05 different from 34 to 38°C*,

# *p < 0.05 different from step, 34 to 38°C*.

**Figure 3:**
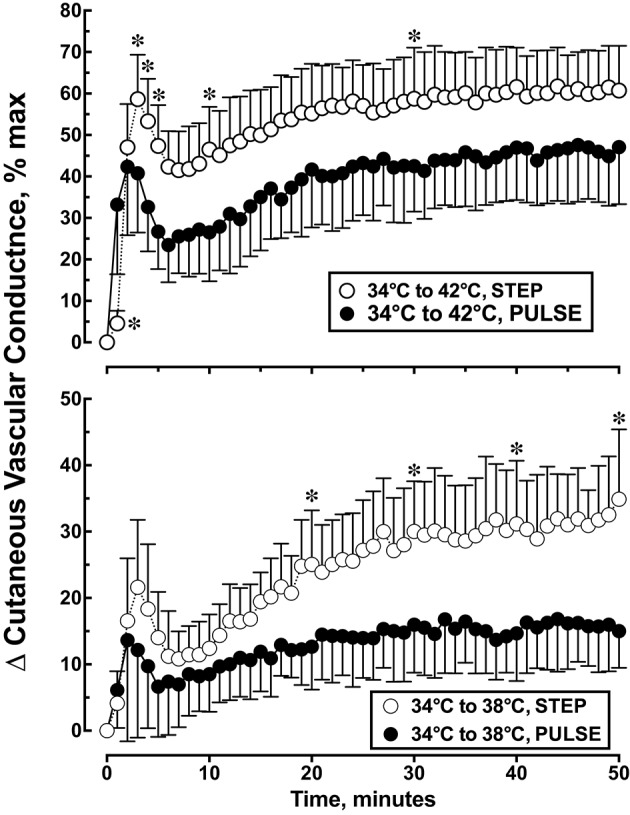
**Cutaneous vascular conductance response to local skin heating during: (1) a single step change in local skin temperature from 34 to 38°C (*n* = 10), (2) a single step change in local skin temperature from 34 to 42°C (*n* = 10), (3) pulsed heating, 1 pulse per min, each pulse represented a step change in local skin temperature from 34 to 38°C, rate of change = 1.0°C • s^−1^, 50% duty cycle, *n* = 10 or (4) pulsed heating, 1 pulse per min, each pulse represented a step change in local skin temperature from 34 to 42°C, rate of change = 1.0°C • s^−1^, 50% duty cycle, *n* = 9**. Values represent the mean ± 1 SD. ^*^p < 0.05 STEP differs from PULSE.

Each local heating protocol produced a biphasic skin blood flow response that was characterized by early peak in CVC, followed by a brief nadir in CVC, and followed by a slow rise to a plateau level by the end of the 50 min heating period. The specific biphasic CVC parameter estimates are listed in Table 1. CVC during the 34°C baseline was similar for all treatment groups and ranged from 20.0 ± 6.6 to 23.8 ± 8.2% CVCmax (*p* = 0.264). Figure 3 illustrates the change in CVC from baseline during each local skin heating protocol. The initial peak CVC response to local heating from 34 to 38°C was similar (*p* = 0.182) for the step change or pulsed heating protocols (Table 1). During local heating from 34 to 42°C initial peak CVC response was lower during pulsed heating (*p* = 0.0065) The plateau CVC response to a skin temperature change of 34 to 38°C or 34 to 42°C was lower with pulsed heating than with a step change in skin temperature (Table 1). In addition, the plateau CVC during pulsed heating from 34 to 42°C was greater than that for the step change in temperature from 34 to 38°C (*p* < 0.05 despite having similar average temperatures.

We estimated the overall CVC response to local heating by calculating the area under the CVC-time curve. *Post-hoc* analysis indicated that the skin temperature challenge of 34 to 38°C, the CVC AUC averaged 1196 ± 295% CVCmax•min during the step change in skin temperature which was 82% larger than the 656 ± 282% CVCmax•min during pulsed heating [*F*_(4, 3)_ = 47.97, *p* < 0.001]. For the skin temperature challenge of 34 to 42°C, the CVC AUC averaged 2678 ± 458% CVCmax•min during the step change in skin temperature which was 37% larger than the 1954 ± 533% CVCmax•min during pulsed heating (*p* < 0.05).

CGRP_dialysate_ at a local skin temperature of 34°C ranged from 3.9 ± 2.1 to 5.7 ± 2.0 pg/ml (Table 2). During local heating protocol CGRP_dialysate_ ranged from 3.6 ± 1.4 to 5.7 ± 1.9 pg/ml. Local skin heating did not produce a significant increase in CGRP_dialysate._

**Table 2:** **CGRP_dialysate_ concentrations from intradermal microdialysis samples**.

**Time period**	**34°C to 38°C**			**34°C to 42°C**		
	**STEP**	**PULSED**	**Cohen's *d* (CI)**	**STEP**	**PULSED**	**Cohen's *d* (CI)**
Baseline (0–25 min)	5.6±1.9	4.7±2.0	0.4 (−0.8, 1.9)	5.5±2.8	3.9±2.1	0.6 (−0.8, 2.1)
Baseline (26–50 min)	5.7±2.0	4.8±1.6	0.5 (−0.9, 1.9)	5.4±1.9	4.8±2.5	0.3 (−1.8, 1.7)
Heat (0–25 min)	5.4±1.7	3.6±1.4	1.0 (−0.4, 2.4)	4.9±1.4	4.0±1.7	0.6 (−0.9, 2.0)
Heat (26–50 min)	5.7±1.9	3.8±1.6	1.1 (0.1, 2.5)	5.1±1.6	3.7±1.7	0.8 (−0.6, 2.3)

*CGRP_dialysate_, concentration in pg • ml^−1^. Values are mean ± 1 SD*.

**Figure 4:**
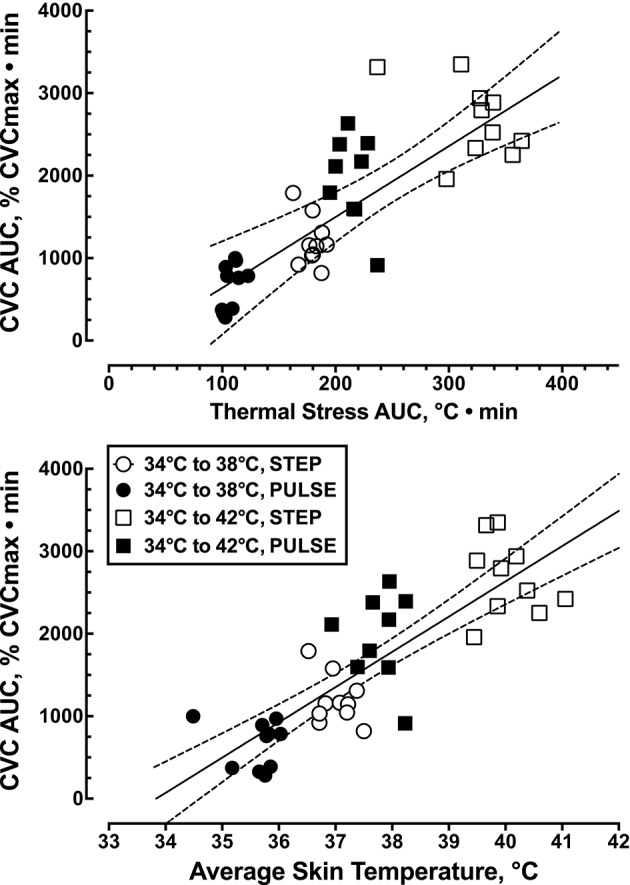
**Relationship between the overall thermal stress applied locally to the skin, expressed as the average skin temperature (*r*^2^ = 0.69, *p* < 0.0001, *n* = 39), and the overall skin blood flow response, expressed as the area under the skin blood flow-time curve (*r*^2^ = 0.62, *p* < 0.0001, *n* = 39)**.

The overall skin blood flow response, expressed as the area under the % CVCmax-time curve was linearly related to the average local thermal stress, estimated from the area under the Tsk-time curve during the entire 50 min local heating period (*r*^2^ = 0.62, *F*_(1, 37)_ = 29.1, *p* < 0.0001, Figure 4). The overall skin blood flow response was also linearly related to the the average skin temperature (*r*^2^ = 0.69, *F*_(1, 37)_ = 82.4, *p* < 0.0001, Figure 4).

**Figure 5:**
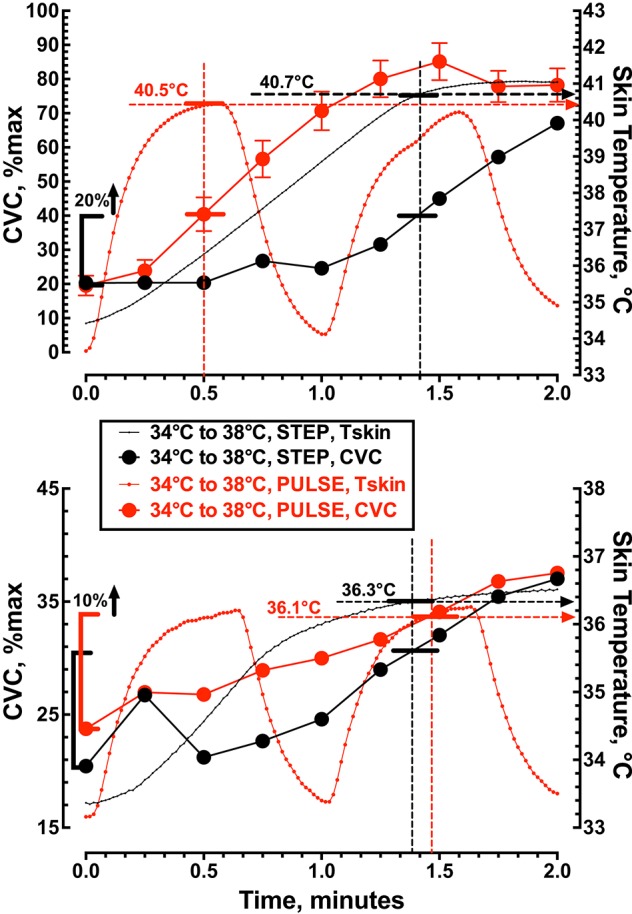
**The relationship between cutaneous vascular conductance and local skin temperature during the first 2 min of local skin heating**. The top panel compares a single step change in local skin temperature from 34 to 42°C (*n* = 10) with pulsed heating of 1 pulse per min, 34 to 42°C, rate of change = 1.0°C • s^−1^, 50% duty cycle (*n* = 9). The lower panel compares a single step change in local skin temperature from 34 to 38°C (*n* = 10) with pulsed heating of 1 pulse per min, 34 to 38°C, rate of change = 1.0°C • s^−1^, 50% duty cycle (*n* = 10). Values represent the mean while the error bars have not been displayed to allow visualization of the analysis.

Figure 5 shows the vasodilator response to a large (34 to 42°C or Δ8°C) and rapid (1°C/s) thermal stimulus (Protocol 4) to the skin that resulted in a local Tskin of ≈40.4°C and a 20% increase in CVC within 30 s. In contrast, the same large (8°C) thermal stimulus (Protocol 2) delivered more slowly (0.1°C/s) increased Tskin to ≈40.7°C during the first 85 s of heating and produced a similar 20% increase in CVC. During a small (34 to 38°C or Δ4°C) rapid (1°C/s) thermal stimulus (Protocol 3) to the skin local Tskin increased to 36.1°C with a 10% increase in CVC within 87 s. The same small (4°C) thermal stimulus (Protocol 1) delivered more slowly (0.1°C/s) increased Tskin to ≈36.3°C during the first 82.5 s of heating and produced a similar 10% increase in CVC. In this analysis local skin temperature was a better predictor of CVC than the area under the Tskin curve.

## Discussion

The magnitude of the vasodilator response to local skin heating was significantly attenuated when the thermal stimuli was delivered in pulses compared to a simple step change in Tsk (Table 1 and Figure 2). The effect size for the reduction in plateau CVC and the area under the % CVCmax- time curve was large (d = 1.2 and −0.9, respectively) indicating that the magnitude of difference was of high practical significance. Warm thermal sensitivity in the skin is mediated by small receptive field warm fibers with a threshold of detection around 33°C. Warm sensitive fibers fire intensely in response to dynamic heat exposure and adapt to short (<60 s) repetitive warm stimulation with a decrease in discharge frequency (Darian-Smith et al., 1979). This discharge pattern is similar to the thermosensitivity of the TRPV4 ion channel (Güler et al., 2002; Schepers and Ringkamp, 2009). The reduced vasodilator response to pulsed heating would be consistent with the expected impact of the pulsed heating protocol on discharge patterns of warm sensory fibers and TRPV4 ion channels. However, the overall thermal load of these two heating protocols were not identical and thus limit this interpretation. We did observe similar thermal loads when comparing a step change in local skin temperature from 34 to 38°C to the pulsed stimulus from 34 to 42°C. Despite similar thermal loads the pulsed temperature stimuli resulted in a significantly larger increase in CVC. However, because the pulse stimulus from 34 to 42°C would have also activated additional TRPV1 channels the comparison of CVC responses may not be appropriate.

The vasomotor response of a large (8°C) and rapid (1°C/s) thermal stimulus (Protocol 4) to the skin produced a 20% increase in CVC within 30 s with a Tskin of 40.4°C (Figure 5). In contrast, a large (8°C) but slow (0.1°C/s) application of the thermal stimulus (Protocol 2) caused a similar 20% increase in CVC that required 85 s with a Tskin of 40.7°C. These data indicate that the initial rise in skin blood flow during local heating depends on the local skin temperature rather than the rate of heating or the ability to activate an axon-mediated vasodilation, presumably mediated by TRPV1 containing sensory nerves. The fact that we saw no increase in CGRP_dialysate_ supports our view that an axon-mediated reflex vasodilation did not contribute to the increase in skin blood flow during the change in local skin temperature from 34 to 38°C. Minson et al. (2001) suggested that blockade of superficial sensory fibers (EMLA cream) reduced the initial rise in skin blood flow by blocking an axon-mediated reflex dilation. In addition, Wong and Fieger (2010) observed a reduction in the initial rise in skin blood flow during local heating when TRPV1 channels were blocked by capsazapine. While we interpret our data to indicate that an axon-reflex mediated release of neuropeptides was not responsible for the initial rise in CVC we cannot exclude the possibility that TRPV1 channels might contribute to the increase in skin blood flow. If TRPV1 channels are involved in the local response to innocuous local skin heating, it may be independent of an axon reflex release of neuropeptides.

Figure 4 illustrates that a large portion of the variation in the overall CVC response to local heating (≈ 67%) could be explained by variation in the level of thermal stress, regardless of the nature of the thermal stimulus (step versus pulsed heating). These data indicate that the cutaneous vasodilator response to local skin heating is primary determined by the magnitude of the local thermal stimulus. As such, these data prevent us from being able to attribute the reduced CVC response during pulsed heating to the desensitization of TRPV4 receptors.

The inability to detect an increase in CGRP_dialysate_ during local heating to 42°C does not support the idea that local thermal stimulus used in our study elicited an axon-mediated reflex dilation. Primary heat nociceptive afferents in the skin are thought to respond to heat via the TRPV1 ion channels (Tominaga and Caterina, 2004) with a threshold temperature of ≈43°C and/or TRPM3 ion channels with a threshold temperature >45°C. Some of these sensory fibers can be activated by capsaicin (Holzer, 1991) and therefore possess heat-sensitive TRPV1 receptors that release neuropeptides (Zimmermann et al., 2005). While “vigorous” or noxious stimuli delivered to the skin can be monitored by these TRPV1 containing neurons (i.e., polymodal nociceptors) and contribute to pain sensation their role in thermosensation is unclear. It has been proposed that the immediate neurogenic response to local skin heating is mediated by activation of primary nociceptive afferents in the skin (Magerl and Treede, 1996). Minson et al. (2001) demonstrated that this immediate neurogenic response could be blunted by application of topical lidocaine to the skin and probably represented an axon mediated reflex. However, lidocaine application to the skin will blunt but not completely block the immediate dilation associated with local heating. Based upon the work of Minson et al. (2001) and Wong and Fieger (2010), our observation of no change in CGRP_dialysate_ was disconcerting. If heating human skin to 42°C activates TRPV1 ion channels the cutaneous vasodilation may not be determined by release of CGRP.

One limitation to this study is the ability of intradermal microdialysis to detect changes in vasoactive neuropeptides in the skin that contribute to cutaneous vasodilation. Weidner et al. (2000) perfused an intradermal microdialysis probe in human skin with 10^−7^ M CGRP and saw only minimal dilation. However, increasing the CGRP concentration in the perfusate to 5.0 × 10^−7^ M produced maximal cutaneous vasodilation. Schmelz et al. (1997), using a hollow fiber probe with a MW cutoff of 300 kD and an *in vitro* probe recovery for CGRP of ≈2%, reported a baseline CGRP_dialysate_ of ≈6.8 pg/ml. In response to a histamine induced axon reflex, CGRP_dialysate_ increased significantly to ≈7.4 pg/ml. Krämer et al. (2005), using similar microdialysis probes, could not detect any CGRP in their dialysate until they added phosphoramidon (a neutral endopeptidase inhibitor) to the perfusate. We report that at a Tsk of 34°C CGRP_dialysate_ averaged ≈ 4.7 pg/ml, using a hollow fiber probe with a MW cut-off of 18 KD and an *in vitro* probe recovery of ≈7%. Since the molecular weight of the neutral endopeptidase is around 93 kD, it is unlikely it crossed our hollow fiber membrane and degraded our sample. However, it is clear that a variety of endopeptidases are active within the dermis (Mentlein and Roos, 1996) that limit accumulation of vasoactive peptides, like CGRP. While we and others have measured interstitial CGRP levels we cannot entirely dismiss the idea that monitoring vasoactive peptide in the dermis by microdialysis is compromised by low recovery rates and the action of endopeptidases. Finally, all the reported levels of interstitial CGRP fall well below the that reported by Weidner et al. (2000) to elicit cutaneous vasodilation, even when correcting for low probe recovery rates.

In summary, the relationship between the local thermal stimulus applied to the skin and the magnitude of the skin blood flow response (Figure 4) illustrates the subtle relationship between local cutaneous thermosensation and cutaneous vasodilation. At present we are left with some conflicting data. First, the ability to attenuate the CVC response to local skin heating with lidocaine or the TRPV1 ion channel blocker capsazepine supports the role of a TRPV1 containing sensory fibers in regulating skin blood flow during local heating (Minson et al., 2001; Wong and Fieger, 2010). However, we were unable to detect any change in the CGRP_dialysate_ during local skin heating. This conflict provides some doubt as to the contribution of neuropeptide release to any subsequent cutaneous vasodilation. However, improved sampling of vasoactive peptides within the dermal interstitial fluid is required in future studies. Our current data on the rapid CVC response to a thermal challenge (Figure 5) clearly shows a potential role for sensory fiber contribution to local heating without the release of significant amounts of neuropeptide. The fact that the overall SkBF response to heating, regardless of the protocol, is a function of the thermal stress applied to the skin points to an integration of local thermosensitive information that is not likely limited to just sensory afferents. Our study failed to clarify the role of temperature-sensitive TRPV4 ion channels in temperature discrimination in human skin. It is likely that multiple redundant molecular temperature sensors (Vriens et al., 2014) are used to evaluate cutaneous thermal stimuli. While we do not know how local thermosensors act to regulate local skin blood flow in humans the present data provides direct support that local cutaneous thermosensation is of critical importance.

## Author contributions

GM: Conception and Design; GM, KF, and WN: Collection and data assembly; GM, KF, and WN: Interpretation of results; GM, KF, and WN: Manuscript writing; GM, KF, and WN: Approval for submission; GM: Financial support.

### Conflict of interest statement

The authors declare that the research was conducted in the absence of any commercial or financial relationships that could be construed as a potential conflict of interest.
